# Adamantinoma Presenting With Local Recurrence and Inguinal Lymph Node Metastasis: A Case Report

**DOI:** 10.7759/cureus.13439

**Published:** 2021-02-19

**Authors:** Martin Zapata Laguado, Eduardo Luis Canales Pacheco, Jesus Oswaldo Sanchez Castillo

**Affiliations:** 1 Clinical Oncology, Universidad el Bosque/Instituto Nacional de Cancerología, Bogota, COL; 2 General Medicine, Universidad del Magdalena/Instituto Nacional de Cancerología, Bogota, COL

**Keywords:** adamantinoma, local recurrence, metastasis, dedifferentiation, lymph nodes

## Abstract

Adamantinoma of long bones is a slow-growing, low-grade primary malignant bone tumor. It is a rare entity and accounts for less than 1% of all primary bone tumors, and in most cases, it occurs in the mid-axis of the tibia of adolescents and young adults. In this report, we describe the case of a 53-year-old woman with a diagnosis of adamantinoma of the left tibia who was initially treated surgically in 2004. Two years later, she presented with local relapse, for which she underwent new surgical management. The patient was referred to our practice 16 years after the initial diagnosis, and she presented with recurrence characterized by ipsilateral inguinal lymph node metastasis. The histological findings and immunohistochemistry were compatible with metastatic adamantinoma with high-grade differentiation, which ultimately required surgical salvage management.

## Introduction

Adamantinoma is a rare bone neoplasm characterized by benign behavior. Usually, it tends to be cured with the first surgical procedure itself. However, there has been anecdotal evidence of its recurrence, which occurs systematically and, in rare instances, locally [1-4]. Regarding its histological differentiation, it is a low-grade and slowly evolving sarcoma. The presence of metastasis is unusual, even though there have been reported cases of relapses at the lung, lymph node, and bone level, which can appear more than 10 years after the diagnosis. In this report, we present the case of a female patient with a diagnosis of adamantinoma of the left lower limb who presented with a relapse at the local and regional ipsilateral inguinal level 16 years after the initial diagnosis. She underwent successful rescue surgical management, after which there has been no evidence of disease progression to date.

## Case presentation

We present the case of a 53-year-old female patient with a history of adamantinoma of the left tibia, which had been managed initially by orthopedic surgery with curative intent in 2004. The patient had presented two years later with local recurrence, and she had undergone wide resection plus allograft implantation at that time. Sixteen years later, the patient was referred to our office with evidence of a new recurrence, documented by the appearance of a new mass in the proximal third of the thigh that extended to the inguinal region, associated with neuropathic pain and Trendelenburg gait due to mass.

Physical exam revealed an indurated mass in the left thigh, which was not painful, had been fixed, and with a diameter of 6 x 6 centimeters. There were no other remarkable findings in the physical exam. The MRI revealed superficial adenopathy in the inguinofemoral region in contact with the muscular fascia and greater saphenous vein, suggestive of secondary compromise as per the first known image. 

A node biopsy was performed, which revealed an immunoprofile that favored the presence of metastatic adamantinoma. The immunochemistry findings were as follows: Enolase +, CD99 +, BCL2 +, P63 +, CKAE1/AE3 focal +, FLI1 +, Synaptophysin -, S100 -, Desmine (-), EMA (-). Abdominal and thorax CT were performed, which revealed the absence of any secondary lesions. 

There was evidence of local compromise, and the patient was scheduled for a salvage procedure, with inguinofemoral lymph node dissection. The chirurgical procedure revealed the presence of spindle cell sarcoma of grade 2-3, with extension to soft tissue and extranodal, coupled with perineural invasion. Mitosis count was as follows: 12 mitosis per camp, no necrosis. Three additional nodes were free of tumor, the skin was free of tumor, and the section edge was also free of tumor. Immunohistochemistry revealed P63 +, p40 +, vimentin +, CK5/6 +, D240 +, EMA focal +, CKAE1AE3 focal +, CAM5.2 focal +.

Due to limited evidence regarding the benefit of chemotherapy or radiation therapy in the adjuvant or “pseudoadjuvant” scenario, and with these procedures thought to be beneficial only in patients with systemical compromise, it was decided to continue with a follow-up strategy for our patient. The patient has remained disease-free for the past six months and has not shown any clinical signs of relapse.

## Discussion

Adamantinoma is a rare primary malignant tumor of the bone that predominantly affects the tibia of young adults, with an incidence rate of less than 1% [1,5,6]. It may appear between the ages of 2-86 years, with a peak incidence rate between the ages of 20-40 years. It is more frequent in men than women [6-8]. It is associated with a good prognosis and high survival rates worldwide, with reported survival rates of 85-95% at five-year follow-ups [9].

The etiology of these tumors is still a matter of debate, but the most widely accepted hypothesis postulates that the displacement of the basal epithelium during the embryologic development, when the bone forms the endochondral surface, generates the precursor cells that give rise to this class of tumor [1]. It has been hypothesized that adamantinoma is of epithelial origin. Based on the immunochemistry and ultrastructural studies, the tumoral cells show strong staining with pan-cytokeratin antibody, and in electron microscopy, the cells have epithelial characteristics such as the basal lamina, desmosome, tight junction, specific epithelial keratin, and a composition similar to that of the epithelial tissue [3].

Surgical management is the preferred treatment modality with en bloc resection with wide margins and limb reconstruction, and it may occasionally lead to amputation based on the extent of bone involvement and the response to initial management. The tumor is highly resistant to radiation, and chemotherapy has not been shown to be effective either [5].

Some studies in the literature have reported that local relapse occurs in 30-35% of cases, with a rate of mortality of 6-18%, and pulmonary metastases or involvement of the lymph nodes in 12-29% of cases [6]. In the paper published by Moon et al., 21 of the patients had 29 sites of clinical metastases at the time of death; 16 of the sites showed pulmonary metastases, and five were found at the lymph nodes [2]. Adamantinoma leads to metastases in 15-30% of the cases by the hematogenous or lymphatic route to other sites but is less frequently associated with the bone and abdominal viscera [2,6].

It has been reported that isolated local recurrence and recurrence in pulmonary parenchyma can occur 13-25 years after the initial diagnosis [4,10]. In the study by Keeney et al., among 85 cases of adamantinoma, 31% of the patients had local recurrence, 15% had pulmonary metastases, and 7% experienced lymph node involvement [6].

There have been scarce reports of local relapse with posterior lymph node compromise, and it is this paucity of such cases that prompted us to discuss this particular case. The common risk factors for local relapses and metastases include the male gender, initial management that is limited to biopsy, positive margins, and narrow margins, with the last one being the main possible cause of the local relapse in the present study [1,4,6,11-13].

In the following table, we present a comparison of the case reports about metastatic adamantinoma; it includes the type of evolution, patient age, histopathological and immunohistochemical classification, as well as the details of the dedifferentiation characteristics noted in our current case.

**Table 1:** Comparative analysis of metastatic adamantinoma cases VEGFR: vascular endothelial growth factor receptor; CK: cytokeratin; EMA: epithelial membrane antigen; CEA: carcinoembryonic antigen; TLE1: transducin-like enhancer of split-1; CD99: cluster of differentiation 99

Study	Primary site	Site of metastasis	Time	Dedifferentiation	Metastasis histology	Immunohistochemistry of metastasis	Gender	Age
Pattabhiraman et al. (2019) [14]	Right tibia	Extensive endobronchial	7 years	No	Metastatic adamantinoma	-	Male	31 years
Cao et al. (2016) [15]	Medial condyle of right femur	Upper lobe right lung	5 years	No	Proliferation of spindle-shaped and squamous epithelial cells with mild nuclear atypia, surrounded by fibrous stroma	-	Female	74 years
Giannoulis et al. (2014) [10]	Right tibia with local recurrence	Left upper lobe and left 6, 7, and 8 ribs	13 years	No	Basaloid cells in a stroma of fibroconnective tissue	Vimentin (+), caldesmon (+), pankeratin (+) (AE1/AE3), calponin (+), and negative to actin of non-striated muscle, to desmin, to CD 117 (c kit), to antigen epithelial membrane, and for S-100	Female	46 years
Flowers et al. (2006) [16]	Left tibia with local recurrence	Bilateral pulmonary	10 months	No	Basaloid and fusiform pattern	CK AE1/AE3 (+), vimentin (+), negative for CK CAM 5.2, EMA, S-100, CEA, or CD99	Male	32 years
Morales Ciancio et al. (2015) [17]	Left tibia with local recurrence	Femur, humerus, lung, L5, and sacrum	5-8 years	High-grade spindle cell sarcoma	Metastatic adamantinoma and dedifferentiation to high-grade spindle cell sarcoma	Positive for CK 5.14 Y19, vimentin, negative for factor VIII, TLE1, and CD99	Male	37 years
Silvestri et al. (2018) [18]	Right tibia	Head of pancreas and lung	1-2 years	No	Metastatic adamantinoma	Anti-CK antibodies AE1 AE3 (+), ActineML (+), vimentin (+), CD99 (+ weak), CD117 (-), VEGFR - 2 (-), PDGFR - beta (-), desmin (-), CK 5/6 (-), S100 (-), and p63 (-)	Female	45 years
Kanakaraddi et al. (2007) [19]	Left tibia	Right femur	4 years	No	Basaloid cells in a fibrous stroma	-	Male	17 years
Panchwagh et al. (2006) [20]	Right tibia	Right femur and lung	6 years	No	Metastatic malignant adamantinoma	-	Female	26 years
Panchwagh et al. (2006) [20]	Left tibia	Left inguinal region	30 years	No	Islands and nests of spindle epithelioid cells with regular hyperchromatic nuclei within a fibromyxoid stroma	Positive for CK AE1/3, CK14, and CK19, focal positivity for CK5/6	Female	53 years

## Conclusions

Adamantinoma is associated with a good prognosis and high overall survival rates; local and distant metastases are rarely reported, which are probably caused by initial surgical management. Surgery is the preferred modality for managing this malignant tumor. A few studies have reported frequent relapses in some cases, and hence it is important to conduct long-term follow-ups for adamantinoma patients.

## References

[1] Turcotte R, Fabbri N (2012). Adamantinoma. EMC - Aparato Locomotor.

[2] Moon NF, Mori H (1986). Adamantinoma of the appendicular skeleton--updated. Clin Orthop Relat Res.

[3] Rosai J, Pinkus GS (1982). Immunohistochemical demonstration of epithelial differentiation in adamantinoma of the tibia. Am J Surg Pathol.

[4] Filippou DK, Papadopoulos V, Kiparidou E, Demertzis NT (2003). Adamantinoma of tibia: a case of late local recurrence along with lung metastases. J Postgrad Med.

[5] Limaiem F, Tafti D, Malik A (2020). Adamantinoma. http://pubmed.ncbi.nlm.nih.gov/30844202/.

[6] Keeney GL, Unni KK, Beabout JW, Pritchard DJ (1989). Adamantinoma of long bones. A clinicopathologic study of 85 cases. Cancer.

[7] Szendroi M, Antal I, Arató G (2009). Adamantinoma of long bones: a long-term follow-up study of 11 cases. Pathol Oncol Res.

[8] Zumárraga JP, Cartolano R, Kohara MT, Baptista AM, Dos Santos FG, de Camargo OP (2018). Tibial adamantinoma: analysis of seven consecutive cases in a single institution. Acta Ortop Bras.

[9] Qureshi AA, Shott S, Mallin BA, Gitelis S (2000). Current trends in the management of adamantinoma of long bones. An international study. J Bone Joint Surg Am.

[10] Giannoulis DK, Gantsos A, Giotis D (2014). Multiple recurrences and late metastasis of adamantinoma in the tibia: a case report. J Orthop Surg (Hong Kong).

[11] Jundt G, Remberger K, Roessner A, Schulz A, Bohndorf K (1995). Adamantinoma of long bones. A histopathological and immunohistochemical study of 23 cases. Pathol Res Pract.

[12] Houdek MT, Sherman CE, Inwards CY, Wenger DE, Rose PS, Sim FH (2018). Adamantinoma of bone: long-term follow-up of 46 consecutive patients. J Surg Oncol.

[13] Ali NM, Niada S, Morris MR, Brini AT, Huen D, Sumathi V, Latif F (2019). Comprehensive molecular characterization of adamantinoma and OFD-like adamantinoma bone tumors. Am J Surg Pathol.

[14] Pattabhiraman VR, Yadav P, Srinivasan A, Sivaramakrishnan M, Shankar A (2019). Interventional management of extensive pulmonary metastasis in adamantinoma. Lung India.

[15] Cao K, Susa M, Watanabe I (2016). Adamantinoma of the distal femur diagnosed 5 years after initial surgery: a case report. J Med Case Rep.

[16] Flowers R, Baliga M, Guo M, Liu SS (2006). Tibial adamantinoma with local recurrence and pulmonary metastasis: report of a case with histocytologic findings. Acta Cytol.

[17] Morales Ciancio RA, Gasbarrini A, Boriani S, Gambarotti M (2015). First confirmed metastatic adamantinoma of the spine: case report and literature review. Global Spine J.

[18] Silvestri S, Deiro G, Sandrucci S, Comandone A, Molinaro L, Chiusa L, Fronda GR, Franchello A (2018). Solitary pancreatic head metastasis from tibial adamantinoma: a rare indication to pancreaticoduodenectomy. J Surg Case Rep.

[19] Kanakaraddi SV, Nagaraj G, Ravinath TM (2007). Adamantinoma of the tibia with late skeletal metastasis: an unusual presentation. J Bone Joint Surg Br.

[20] Panchwagh Y, Puri A, Agarwal M, Chinoy R, Jambhekar N (2006). Case report: metastatic adamantinoma of the tibia--an unusual presentation. Skeletal Radiol.

